# Teen Girls’ Experiences Negotiating the Ubiquitous Dick Pic: Sexual Double Standards and the Normalization of Image Based Sexual Harassment

**DOI:** 10.1007/s11199-021-01236-3

**Published:** 2021-07-23

**Authors:** Jessica Ringrose, Kaitlyn Regehr, Sophie Whitehead

**Affiliations:** 1 UCL Institute of Education, 20 Bedford Way, London, WC1H 0AL UK; 2 University of Kent, Jarman Building (Office 2.45), Canterbury, CT2 7UG UK; 3 Digital Humanities, Kings College London, Chesham Building, Strand, London, WC2R 2LS UK

**Keywords:** Sexting, Dick pics, Adolescents, Social media, Image-based sexual harassment, Sexual double standards, Focus groups, Arts based qualitative methods

## Abstract

A range of important studies have recently explored adult women’s experiences of receiving unwanted dick pics (Amundsen, 2020). However, to date there has been limited research that has explored teen girls’ experiences of receiving unwanted penis images in depth. To address this gap we draw upon our findings from a qualitative study using focus group interviews and arts based drawing methods to explore social media image sharing practices with 144 young people aged 11–18 in seven secondary schools in England. We argue that being bombarded with unwanted dick pics on social media platforms like Snapchat normalises harassing practices as signs of desirability and popularity for girls, but suggest that being sent unsolicited dick pics from boys at school is more difficult for girls to manage or report than ignoring or blocking random older senders. We also found that due to sexual double standards girls were not able to leverage dick pics for status in the same way boys can use girls’ nudes as social currency, since girls faced the possibility of being shamed for being known recipients of dick pics. Finally we explore how some girls challenge abusive elements of toxic masculinity in the drawing sessions and our conclusion argues that unwanted dick pics should always be understood as forms of image based sexual harassment.

Nearly a decade ago, Ringrose et al. (2012) conducted one of the first qualitative studies on youth sexting in the UK. The research sought to explore the gendered discourses, logics, and rationales behind young people’s practices of networked sexual image exchange; particularly how normative and gender binary ideals of femininity and masculinity led to pressures for girls to produce and share sexual images of themselves, much more than boys (see also Setty, 2019a). Through further research with a range of international collaborators we also sought to deconstruct a misogynistic shaming and victim-blaming dynamic in school sexting education cultures and teaching resources like anti-sexting films (Dobson & Ringrose, 2016) by pinpointing and illuminating the deeply sexist social exchange logics behind the images. We found that the images were valuable to boys as currency because they denoted ownership over girls’ bodies (Ringrose & Harvey, 2015) thus rendering girls’ bodies interchangeable parts to be traded (Dodge, 2020; Ringrose et al., 2013).

At the time of that research, also speaking to young people of secondary school age, we explored pressure upon boys to ask girls for sexual images (Ringrose et al., 2013), and then to prove they had garnered these by showing and sharing them as a part of a "homosocial reward" dynamic in which status correlated with heteronormative masculine sexuality (Ringrose & Harvey, 2015, p. 206). We did not however spend much time discussing nude images sent by boys; as they featured less in the findings. They did appear when boys could capture evidence of "blow jobs" for instance, but the practice of randomly sending one’s penis uninvited (colloquially known as sending a dick pic) was still relatively rare in the research data collected in 2011 (Ringrose et al., 2012). Now, a decade later, it appears that the central shift that has occurred in the relational dynamics of youth sexting is the normalisation of the dick pic in the social media ecosystems of young people (Ricciardelli & Adorjan, 2018).

In this paper, we explore girls’ experiences of receiving dick pics across seven diverse secondary schools in the UK. We focus in depth on adolescent girls’ experiences and understandings of receiving dick pics, as there has been limited research on this age group (Madigan et al., 2018). We start by exploring the receiving of unwanted dick pics from predatory older men on Snapchat, which we argue normalizes this content but also has features of being unknown and random and therefore relatively easy to ignore or block. Next, we look at a range of practices when dick pics are sent from networked peers in the girls’ age range. These include faceless, fake and accidental dick pics as well images from "friends of friends" or peers known only online. We investigate how and why unwanted receipt of dick pics from known boys at school can create very difficult dynamics for girls to navigate and the challenges they face in reporting these images. We also consider how not all uninvited dick pics are viewed negatively. In some cases, receiving unsolicited dick pics has become understood as a badge of desire. Further some girls ask for penis images but can face sexual stigma and shame for doing so because of sexual double standards.

In our final section of data analysis, we reflect on how the drawing methodologies created spaces for girls to engage with and sometimes challenge problematic social media practices of masculinity and lad banter surrounding penis images. We conclude the paper by outlining the limitations of the research and implications for youth image sharing practices, recommending a shift in how we understand and respond to unwanted dick pics to disrupt their normalisation and now ubiquitous nature (Ricciardelli & Adorjan, 2018). We argue dick pics must always be understood through dynamics of consent and when they are unwanted they need to be reconceptualised as forms of image based sexual harassment (McGlynn & Johnson, 2020).

## Policy and Research Context

At present, in the UK adult men sending dick pics to under 18 s is categorized as indecent and a sexual offence, but there is a lack of awareness of the practice (BBC News, 2018). According to the UK Council for Child Internet Safety (2016), minors under 18 years of age producing images of themselves is, however, an illegal act. The fact that nude images of under 18 s constitute child pornography has been the focus of most government guidance and educational resources, with an abstinence message to youth to stop sexting. Government and school policy around sexting has mostly focused on girls as those assumed to be taking and sending more images, although in some cases resources have looked at the dangers of primary-aged boys producing images of their penises (National Society for the Prevention of Cruelty to Children, 2016). The masculinity practices of men and boys sending images of their penises as acts of online harassment and girls experiences of receiving such images has, however, been largely neglected in official guidance for young people in the UK (Department for Education, 2020).

In contrast to the lack of educational awareness and focused guidance on unsolicited dick pics in the UK, a range of research demonstrates this is a growing social problem for girls and women. In a 2018 a UK YouGov poll of more than 2000 women, of those aged between 18–24 years of age, 54% had received a dick pic, with 47% of that figure, being unsolicited (Smith, 2018). The YouGov poll also highlighted that, of all of the women questioned, 46% received an unsolicited dick pic before the age of 18; a sexual offence under UK law (Smith, 2018). International academic literature has suggested that dick pics sent to women, especially young women, are often unsolicited and sent from heterosexual, cisgender men (Waling & Pym, 2019). Waling and Pym highlight that there has been much public commentary on the dick pic which centres around ideas that senders are either clueless about what recipients might find attractive, or that senders share dick pics with the hope of receiving an image in return. Waling and Pym call urgently for further empirical research on experiences of dick pics from the perspective of both senders and recipients.

Ricciardelli and Adorjan (2018) have explored the gendered double standards in youth sexting that have led to the normalisation of non-consensual dick pics. In their research with 115 teenagers aged 13–19 in Canada; it was so common for girls to receive unsolicited dick pics they joked that they were going to create a scrapbook to commemorate them at the end of grade 12 (although this was a passing comment it did partly inspire us around our participatory drawing methodology to capture the experiences of receiving dick pics). Hayes and Dragiewicz (2018) analyzing the rise of dick pics and their normalisation discuss the omissions in the law regarding this practice. They argue this has led to difficulties in responding given that "flashing" offline is a criminal offence whereas cyber flashing online is not; and question why dick pics are not taken more seriously in the online sphere. Hayes and Dragiewicz (2018, p. 116) use Liz Kelly’s (1998) continuum of sexual abuse to position receiving unsolicited dick pics—the act of sending one’s penis without being asked (without consent)—as an expression of masculine sexual entitlement and of the exertion of male power. Legal scholars Clare McGlynn and Johnson argue similarly that all unwanted penis images should be criminalised as *cyberflashing*, and Powell and Henry (2017) position unwanted dick pics as a form of technologically facilitated sexual violence and image based sexual harassment, something which we consider in our conclusions.

Oswald et al. (2019) completed a survey study with 1,087 adult participants which found the primary motive for sending a dick pic was to receive an image in return, something that girls in our study also confirm. Subsequent reasons included the hope of sexually arousing the recipient and, for a smaller percentage, misogyny, power and control were an overt element of their motivation. Mandau’s (2020) qualitative research finds that boys and men (aged 17–20 in the study) perceive “the sending of dick pics as a way of showing off, complimenting, hooking-up with or getting nudes in return from girls” (p. 72). Further research has looked at transactional sexting and dick pics sent in an explicit bid to solicit nudes back from girls (Ravn et al., 2019). We will explore how this practice is a form of *doubled harassment* when dick pics are unwanted but also part of pressuring girls to send nudes back (Englander & McCoy, 2016). Salter (2016a) asserts that men’s penises are deployed as a form of online sexual harassment, and yet girls and women were typically held responsible for managing online risks. Amundsen’s (2020) findings showed similarly that adult women in her study adopted individualised strategies of ignoring and blocking senders. The women positioned dick pics as a form of sexism but they typically did not understand these as online harassment. Amundsen found that post-feminist notions of individualised choice and responsibilization of women’s experiences of risk and harm in sexual encounters (something endemic to rape culture) meant that the adult women did not view these practices as abusive and did not want to position themselves as victims. This is a critical trend that we observe in our data where masculine aggression is naturalized and normalised, which “obscures from view the unequal and highly gendered social structures that both grant unsolicited DPs [dick pics] their harmful meanings and make such sexist practices possible from the outset” (Amundsen, 2020, p. 5).

Of course, not all dick pics sent to girls and young women are non-consensual and unwanted (Naezer & van Oosterhout, 2021). Hayes and Dragiweicz (2018) discuss other frames of understanding dick pics as erotica and part of courtship rituals amongst adults. A great deal of previous research has found, however, that girls who are open about sexting can be branded as slutty, because of sexual double standards (Naezer & van Oosterhout, 2021; Ravn et al., 2019; Ringrose et al., 2013). We will explore practices of girls asking for and keeping dick pics and girls’ negotiation of sexual double standards and shaming. In addition, Vitis and Gilmour (2017) explored ways of resisting the harassing dynamics of unsolicited dick pics and sexism online, by using art and humorous language to reverse shame back onto dick pick perpetrators. Similarly, Ringrose and Lawrence (2018) examined a Tumblr site where men submit dick pics for ratings posted by the site host, who uses humour to create scales of value of the dick pics, which subverts and reworks the force relations of the penis images. We consider possibilities of disrupting narratives of feminine passivity and vulnerability around receiving dick pics, particularly through our analysis of the girls’ drawing sessions. Overall, there has been very little research that has looked in depth at adolescent girls’ experiences of receiving dick pics; most research is with adults (Amundsen, 2020) or those aged 17 and above (Mandau, 2020) and/or there is limited discussion of girls’ experiences as recipients of dick pics presented in the findings (Naezer & van Oosterhout, 2021; Ravn et al., 2019; Ricciardelli & Adorjan, 2018; Setty, 2019a). We, therefore, focus on this element in our analysis to fill a gap in understanding about how girls respond to and manage being sent dick pics.

## Method

In 2019 we undertook research in seven highly diverse secondary schools using focus group discussions and participatory arts-based methods of social media post drawing to recreate experiences of sharing and receiving digital sexual images. As can be seen in Table 1, four of these schools were mixed state comprehensives; and three were independent schools (‘single sex’ boy, girl and mixed boarding). Prior to data collection, we obtained full ethical approval for our project from the University Research Ethics Advisory Group. For all participants 16 years and younger, we obtained parental consent to participate in the research. We worked with 144 young people aged 11–18. Most participants were under 15 years old, creating a unique data set with children under the age of sexual consent (16) and under the legal sexting age (18).

**Table 1:** Breakdown of Research Sites and Participants by Year Groups and Genders

School name	School type	Location	Year groups	Genders
South East London Community School – (SELC)	Mixed state secondary	South East London	Year 8 Year 10 Year 10	12 Mixed (4 boys, 8 girls) 7 girls 6 boys
North East London Academy – (NELA)	Mixed state secondary	North East London	Year 7 Year 8 Year 9 Year 10	7 Mixed 6 girls 1 Gender fluid 3 girls 2 girls 5 Mixed 2 girls, 3 boys
Central London Mixed Comprehensive One (CLC1)	Mixed state secondary	Central London	Year 7 Year 7 Year 9 Year 9	5 boys 8 girls 6 girls 6 boys
Central London Mixed Comprehensive Two (CLC 2)	Mixed state secondary	Central London	Year 9 Year 9 Year 10 Year 10	4 girls 3 boys 4 girls 2 boys
Swans Independent School for Girls	Girls independent with mixed 6^th^ form	South West England	Year 8 Year 9 Year 10 Year 12	8 girls 8 girls 8 girls 8 Mixed (5 girls, 3 boys)
Lords Independent School for Boys	Boys independent	North London	Year 9 Year 9 Year 10 Year 10	3 boys 3 boys 4 boys 3 boys
South East Independent Boarding School (SEI Boarding)	Mixed independent	South East England	Year 8 Year 8	8 girls 10 boys

**Fig. 1:**
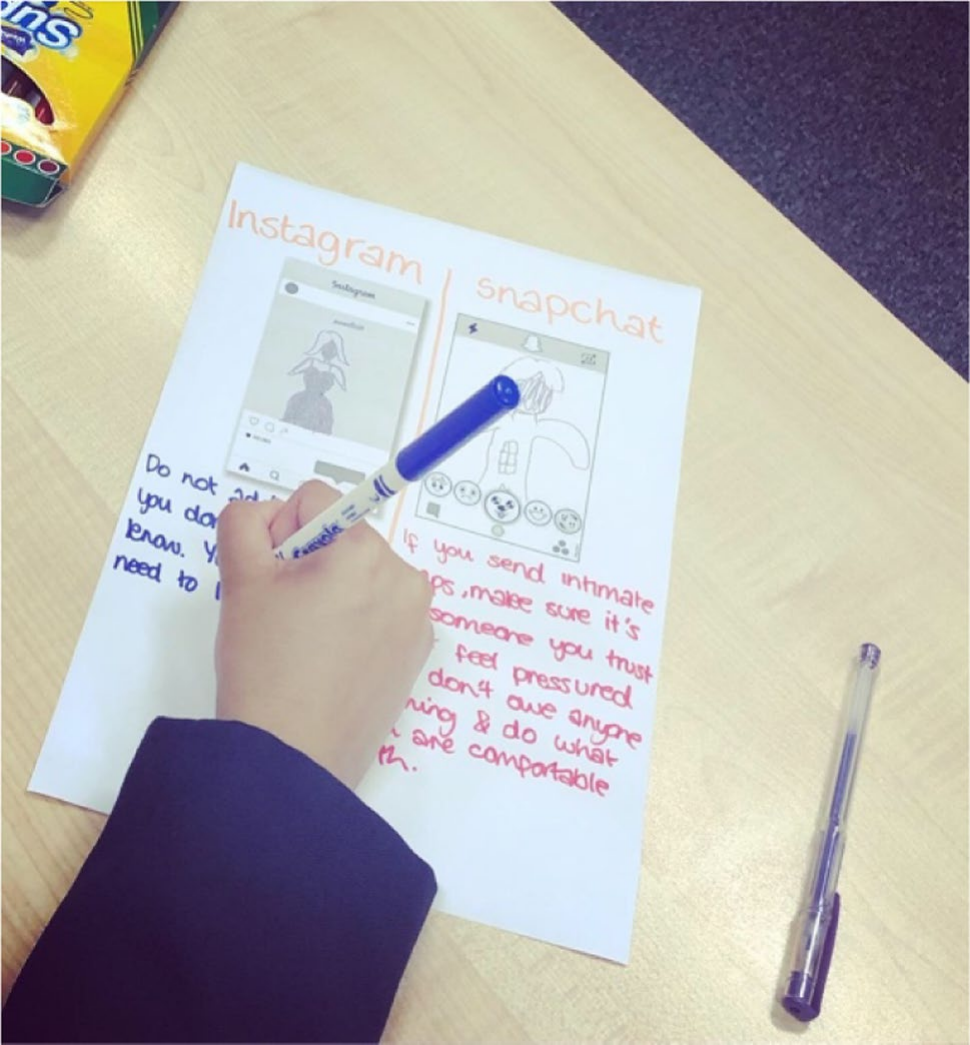
Instagram and Snapchat mAPPing drawing exercise. (CLC2, Year 10 girls)

The Sharing Networked Images Practices (SNIP) mAPPing workshops were developed to map the relational pathways of sexual images and how they are both understood, but also produced—taken, shared and received by young people, through drawing exercises about social media image based applications (hence the APP in mAPPing). First, we used visual prompts of celebrity selfies to generate discussion about norms and rules around taking and sharing images of self and others (Warfield, 2016). Following on from the discussion part of the focus group we then asked young people to draw some of the experiences they had discussed. We drew on Venema and Lobinger’s (2017) use of participatory drawing, where they asked participants to create *relational maps* of how they share images online and where and with who. Adapting this methodology to the youth context, we wanted to explore which platforms young people used to share images, which type of images they received, and whether they wanted them or not. We provided paper templates that showed blank display screens to facilitate drawing activities about Snapchat, Instagram, Youtube, WhatsApp, Facebook and Pornhub. However, through our first few sessions, we learned that the dominant social media platforms for receiving nudes, were Snapchat and Instagram and most drawings focused on using these templates (see Fig. 1).

This opportunity to create visual data provided a mechanism for the participants to draw and quite literally, *show* and tell their experiences of receiving and sharing images in ways that offered up different understandings than interview talk. The drawing methodology enabled us to explore the specificity of content shared and received as well as capture disappearing media like Snapchat images (which there is often no record of). We also asked young people to creatively respond to any issues raised in discussion or their drawings by presenting top tips for better digital sex education, and they created mind maps, lists and diagrams as we’ll explore. All interview and drawing data is anonymized and pseudonyms are used throughout.

Analytically, we use a feminist discursive-material lens to explore how power and gender are operating in sexual image exchange practices with material effects/affects around specific body parts (Lazar, 2005; Renold & Ringrose, 2016; Warfield, 2016). It is important to also qualify that we approach the category of gender critically, we do not essentialise gender, and we did have several non-binary gender participants although most identified as either girl or boy (Bragg et al., 2018). In our analysis of drawings, we mostly discuss penis images, but in a digital context we cannot confirm whether the images were sent by a boy or a man, and indeed we do discuss the phenomena of fake dick pics (Salter, 2016a). We are also focusing on cisgender heterosexual norms in youth digital sexual cultures. Given most of our sample was under 15 years of age, we only had a few young people who openly identified as LGBTQ. Moreover, we are focusing on girls experiences of receiving dick pics in this paper, but we look at masculinity practices and boys’ experiences of youth digital sexual intimacies elsewhere (see Ringrose et al., 2021).

## Results

### Girls’ Experiences of Receiving Random Anonymous Dick Pics

Although this was a qualitative study, we did ask all groups if they had received unwanted nudes from boys through a show of hands in each focus group and found seventy-six per cent of girls had received a dick pic. This number is particularly high because of the in-depth discussions (lasting up to 2 hours) we engaged in that explored their experiences through talk and drawing. Girls typically said that the dick pics they received were "not asked for" or "wanted," but they did not feel they could report them to the school or the online platform. We also found only a slightly lower percentage (70%) of girls had been asked to send nude pics, however, many times this was via being sent an unsolicited dick pic to initiate "trades," which has been referred to as transactional dick pics (Ravn et al., 2019). Girls’ responses typically included variations of the following refrains: *Reporting is hard. Ignoring is better. Blocking is easier, but may not work*. These key themes around girls’ experiences of receiving dick pics echoed throughout this project. While the majority of stories we will consider involve known or semi-known senders, such as friends or boys known at school or in peer network, it is important to point out that the majority of unsolicited dick pics first received by participants came from unknown senders. We argue that it is the frequency of this phenomenon that consequently leads to the normalisation of dick pics from known senders too.

#### Blocking is Easier

Exploring the affective economies of social media platforms, it is crucial to acknowledge the platform affordances (Boyd, 2010), that is the technical functions through which the platform works and which create the conditions of possibility for communication. Snapchat is a platform famous for disappearing and ephemeral content images and/or video, which is exchanged in the form of "streaks" between users (where you keep the streak alive by responding back and forth within a 24-hour period) (Charteris et al., 2018; Handyside & Ringrose, 2017; Koefed & Larson, 2016). Snapchat also has a quick add contact function where unknown contacts can be quickly added. For young people, this function was often associated with using a ‘shout out’ function on Snapchat where messages about a contact can be shared in a bid for more contacts, as this would increase their snapchat user scores. Heavy Snapchat users counted these scores and kept their accounts open to quick adds, which meant they turned off any privacy settings. This then sets the conditions for adults to easily identify children’s networks and infiltrate them. For instance, in discussing retrospectively the first time that young people received dick pics, it was almost unanimously on Snapchat without privacy functions enabled:

Interviewer: When was the first time you got an unwanted dick pic?

Camilla: Probably when we got Snapchat.

Helen: Yeah, literally when we got Snapchat.

(Swans, Year 12 mixed)

These 17 and 18-year-olds are reflecting back to three years ago when they were 15, but the same dynamics were discussed as happening much earlier in other schools, with Year 7 and Year 8 girls explaining that random people added them on Snapchat since they joined in Year 6 (10–11 years old). The exchange below was typical of conversations we had with girls about their experiences of dick pics and "grown men" on Snapchat:

Annie: Snapchat you get sent loads of different things…

Sonja: Yeah.

Annie: By people that you don’t know.

Manjit: …that you don’t want to experience at this age.

Corri: So, the best thing you can do is block them.

Danni: Grown men as well.

Sonja: Yeah.

Annie: Not children, grown men.

Danni: Add you on Snapchat, send it [dick pic] to you, ask for one back.

Manjit: Yeah, or videos.

(NELA, Year s7 mixed)

**Fig. 2:**
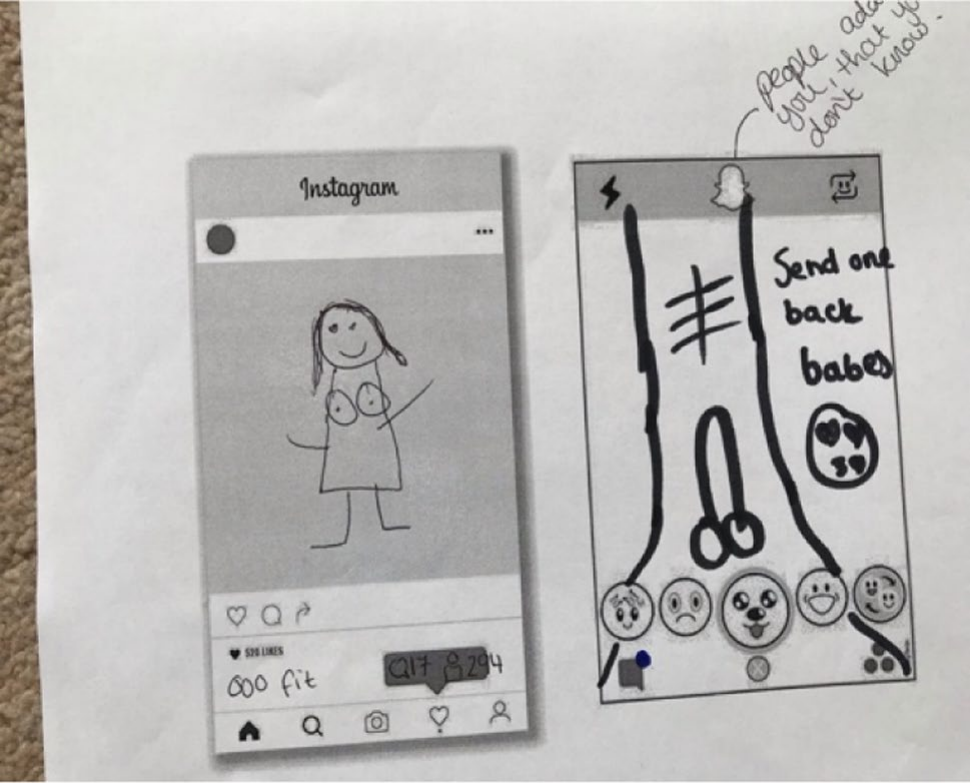
Drawing of Snapchat dick pic trade initiation: “Send one back babes” (NELA, Year 7 mixed group)

Figure 2 documents a Year 7, 12-year-old girl receiving a dick pick invitation to trade on Snapchat*.*

Another Year 8 girl told us about being sat next to her nan and dad, watching TV when she received a dick pic:

I was at my nan’s house… because my nan watches Pointless, and stuff like that, and I can’t be arsed to watch Pointless so I was just on my phone. I was scared. My dad was sitting next to me, so I was just like…LAUGHS… Yeah, like I don’t want mum to see this. I cried. I was swearing down the phone, so uncomfortable. (SELC, Year 8 mixed)

Similarly, a group of Year 8 girls discussed how senders may pose as young people to send images:

Carrie: Someone, yesterday someone was like to me… oh you’re cute, do you wanna see a picture of me? And I said no, how old are you? They said fifteen, and they sent a picture and they looked like forty-nine. And then I just blocked them…

Interviewer: Has everyone in this room been sent a dick pic?

Carrie: Yeah.

Ash: Yeah.

Shanice: I blocked a lot of people on Snapchat.

Ash: Same.

Carrie: I just kept them [the contacts] and they send streaks to me but they masturbate while sending streaks.

(CLC1, Year 8 girls)

**Fig. 3:**
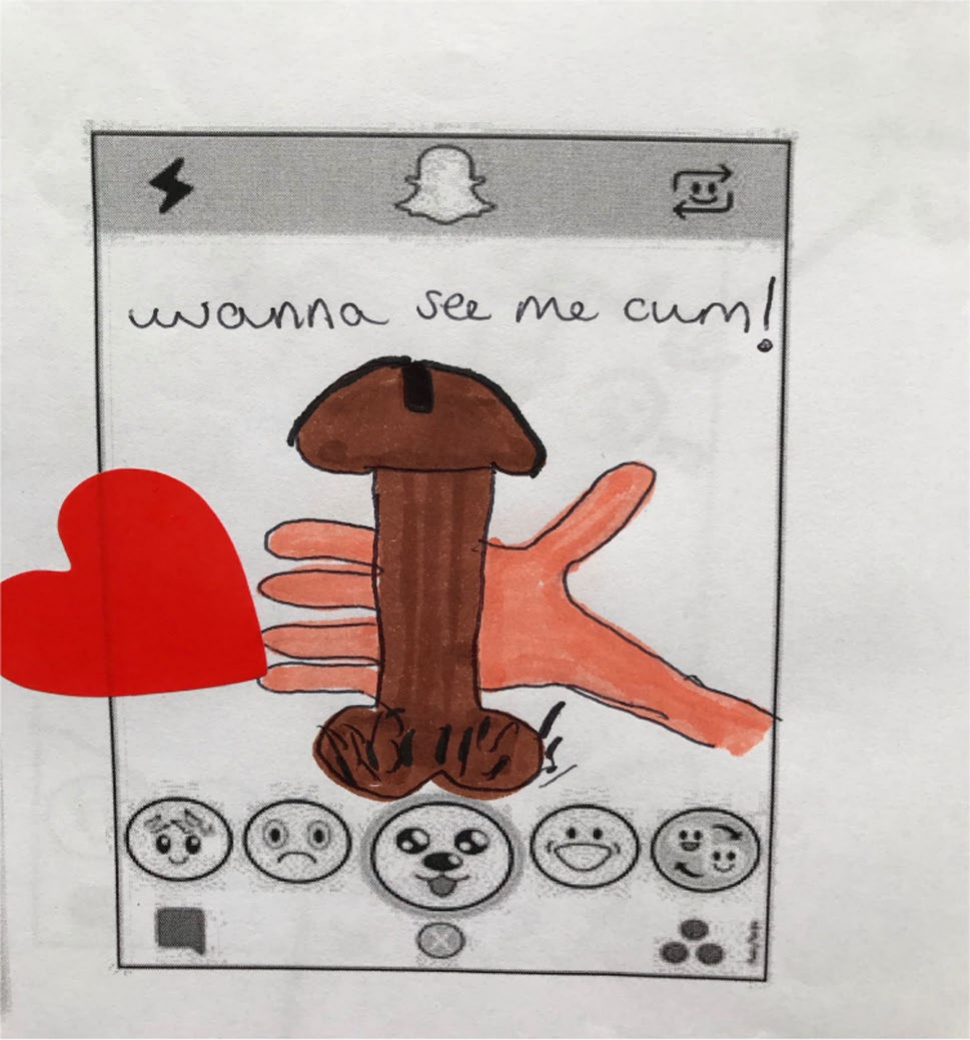
Drawing of a masturbation streak on Snapchat: “wanna see me cum!” (CLC1, Year 8 girls)

Figure 3 documents a masturbation streak on Snapchat.

The social capital of the Snapchat streak in this peer group functions as a tool for older men to access the accounts of young people. Carrie stated further that she is “here [Snapchat] to do streaks and get a high score.” The game-like functionality of Snapchat means young people want high streak scores and so therefore accept unknown contacts, which the young people then have to consistently block. This demonstrates the way in which certain platforms, which are ostensibly innocuous, can serve to facilitate online sexual harassment in unforeseen ways, supporting Chateris et al.’s (2018) claim that educators ought to be aware of the specific affordances of the platforms used by young people.

#### Ignoring is Better

Another group of Year 10 girls said even if their account is on private with the quick add function someone can be added who is a friend of a friend. One girl got 60 adds in one minute after a Snapchat shout out, attracting a "grown man" she then had to block:

Alexus: So, like my account’s on private, but say people add me, I’ll just add them back, so I don’t really pay attention to who’s adding me… there was this one man and he was texting me for months, …and I was ignoring it, like I’d half slide it and then just ignore it… so I was opening like streaks and I just saw it, I didn’t know what it was at first because it was unclear, so I replayed the video and the person must have thought that I liked it or something, because he kept texting me after that, then I was thinking what are you doing? Because it was a grown man. And then I just blocked him afterwards because I was disgusted…It was him like masturbating and doing stuff with his dick, I couldn’t think of the word for it, yeah, and then he showed his face in the video as well.

(SELC, Year 10 girl)

One of the most significant findings about the barrage of dick pics and masturbation videos from older men on Snapchat was the strategies that girls had developed to manage this experience. Where at-first they found the content "disgusting," they quickly adapted to this and became normalised to these dynamics using techniques like Alexus “half sliding” videos (not fully opening the message) in order to not register interest to the sender. They also managed bombardment through a process of ignoring or finally blocking. They said they rarely reported on Snapchat because nothing would be done since images had largely disappeared and instead, they discussed the feeling of having to get on with their life:

Maddie: I accepted this follow request and then on his story it was like “who wants to see my big…you know,” and then I saw like a text from him, because you know you … when I pressed on it, it was a picture of his like dick,…And [girls] they just kind of get used to it after a while (Swans, Year 9 girls)

Helen: But it’s so common, it’s not shocking anymore, you just get on with your life, you’re like yeah.

Amy: It’s just like another one.

Camilla: Laugh and then you carry on.

(Swans, Year 12 mixed)

Lizzie: It just depends, some people are more casual about it than other people.

Alice: Some people will be like…Ugh, it’s a dick pic.

Claire: I think with nudes it’s like drugs, everyone knows it’s bad but they want to do it anyway.

(Swans, Year 8 girls)

A dynamic around dick pic as normal and to be expected is apparent across three of the age groups in Swans School. The Year 9 girls discussed “getting used to it” and the Year 12 girls confirmed that dick pics are just a part of life now that you laugh and “carry on,” and the Year 8 girls suggested some people get “more casual” about dick pics, and despite “everyone” knowing nudes are bad and “like drugs” (signalling an awareness of illegality), “they want to do it anyway.” Here, the bombardment and ubiquity of the unwanted images on Snapchat sets the scene for their normalisation. In the following section, we will delve into the dynamics around being sent and managing dick pics from same-age range boys and known boys from school. We will explore how managing peer group relations are much more complex and potentially difficult than dealing with the content from the unknown random older “paedos.”

### Receiving Unwanted Dick Pics from Peers

#### Transactional Dick Pick Initiations

A critical element of receiving dick pics from peers in the same age range is that these exchanges are often positioned as transactional. That is, boys will often send an image to elicit a nude pic back from the girl rather than seeking to provide sexual pleasure for the girl:

Jada: I had a friend, yeah, and her boyfriend must have sent her a dick pic, and then he carried on trying to pressure her to send one, I feel that’s what happens the most, these boys try and pressure them like into sending it back, because oh I send, or oh if you love me you’ll send it back to me.

Nia: Yeah, if you don’t want me to break up with you, or something like that.

Alexus: They’ll send one and be like now it’s your turn.

Nia: That’s the worst one.

Interviewer: On that how often do you think dick pics are sent with the aim of getting something back?

Rianna: All the time.

Nia: All the time.

Jada: All the time.

Nia: That’s the main point of it, they don’t do it and just be like ‘enjoy’.

[LAUGHTER]

Alexus: They’ll want one back.

(SELC, Year 10 girls)

**Fig. 4:**
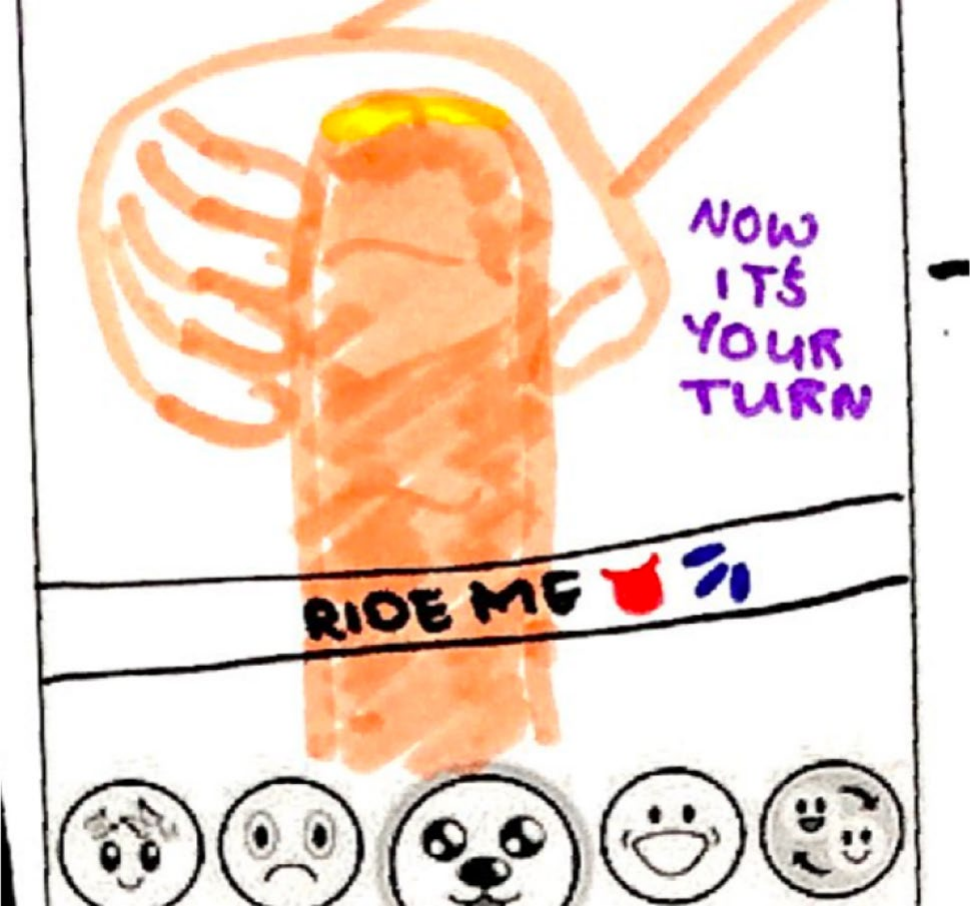
Drawing of a Dick pic sent to initiate a trade for a girl’s nude: “Ride me: Now it's your turn” (CLC1, Year 10 girls)

Figure 4 is of a transactional invitation dick pic to “ride me” and send one back.

The girls explained that when teenage boys they know send dick pics it is typically a transactional bid to get a nude back from the girls. The ubiquity of these exchanges, where girls come to expect messages, and are pressured to participate in an exchange of images, can be seen in the above exchange where they repeat being propositioned with “now it’s your turn” and it happens “all the time.”

Some participants described boys sending *fake* dick pics to girls in order to position the request for nudes as a transactional "trade," without having to actually send their own pic:

Carrie: I know some people go on Google Images and just find one that matches your skin tone, so don’t actually send your own one, and then they get something back.

Shanice: That’s happened before.

Sam: People have Photoshop, they can do anything, they can take a picture of their hand and put it on there.

Ash: People as well, they go on Google and then they look for naked bodies of people, and then they post it on Snapchat and say oh this is someone, when it’s not even them.

(CLC1, Year e8 girls)

As mentioned above, girls also commented on the affordance of the dick pic as typically concealing the face of the boy:

Julia: I’ve actually had a couple of boys, yeah, that haven’t felt comfortable sending a picture of their face, but felt more comfortable sending pictures of their dick. And that was a bit weird.

(CLC1, Year 10 girls)

**Fig. 5:**
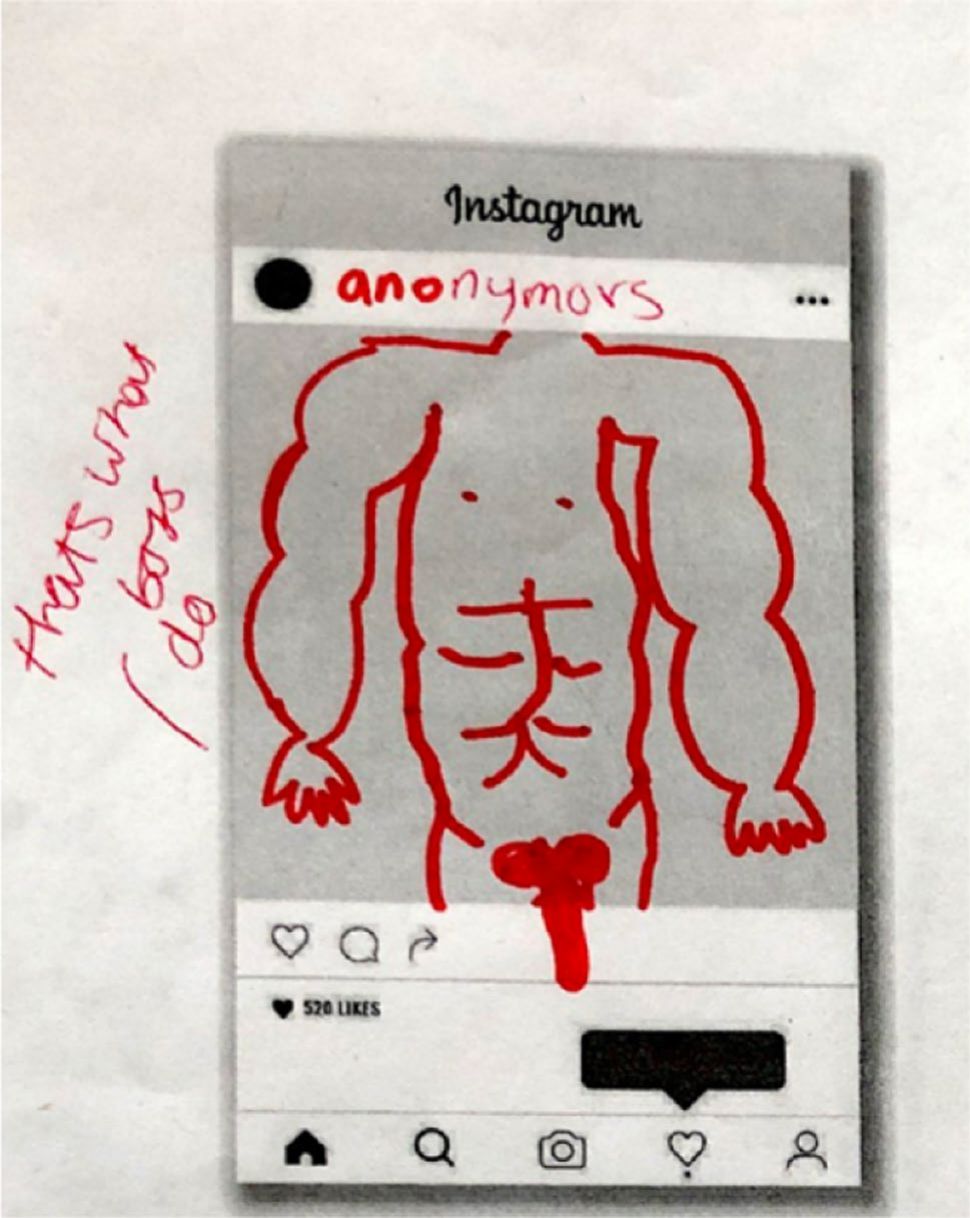
Drawing of a faceless dick pic on Instagram: “Anonymous: that's what boys do” (Swans, Year 9 girls)

The differential power relations around the boys’ dick pic vs. girls’ nudes are evident in part because dick pics often do not include the face, and therefore connecting them to the sender may not be possible. This element of the faceless dick pic was significant in that meant it was difficult to actually report senders. Figure 5 shows a boy’s nude with the face cut off, noting, “that’s what boys do.”

####  “Friend of a Friend”: Dick Pics from Peers

In addition to the often, faceless nature of the dick pics, girls described it being common for there to be some degree of separation from their immediate school or peer group, noting it is more common to receive dick pics from a friend of a friend in their social media networks, rather than boys at school, whom they know directly. Girls suggested it would often be boys from a neighbouring school who sent the images in order to have some degree of distance:

Kiara: …it's the same story like I don't know them. It's just boys from the other school send us both one.

Molly: It's another, it's another secondary school…it's like right next to our school yeah.

Interviewer: So, they've obviously seen you or?

Molly: I think they know people from here and they're trying our usernames.

Kiara: Because when people tag you in a post or whatever like I've seen her around or like I like I know her I know a friend or something like that then they'll be like I'll add them and start talking to them.

(NELA, Year 10 girls)

The visibility of usernames via Snapchat affordances and posts, and being linked to a school based online friend group, facilitates a culture in which social interaction operates across a network of semi-known/unknown actors. Contacts have the guise of familiarity because of inter-school connections, yet, still benefit from online anonymity. The indistinct status of these online users means they cannot be categorised as either stranger or friend—instead existing in a hazy, indeterminate group. The combination of familiarity and anonymity means that users can send dick pics with ease by accessing girls within a broad network of semi-known contacts, typically with minimal risk for the sender.

### Reporting is Hard

#### Getting Dick Picked Stigmatizes Girls

Friend of friend stories were common in the elite schools where networked cultures developed between girls’ schools and local boys’ schools, and online spaces were often the only place to meet boys. In the elite girls’ school, a high-status boy (his father was a known politician) who was “friends” with a peer from a neighbouring boys school had sent dick pics to a girl, and then tried to “force” her to send nudes back:

Lizzie: So I was dating this boy, he said he was thirteen… and then his best friend told me he’s turning fifteen… but then he used to send me nudes, and I didn’t really like him as well, and he was also… he used to force me, but I didn’t send, but everyone thought I did send him, and these rumours all spread around...Everyone told that I sent nudes to him, but I never did. Because everyone was thinking like oh if he sent nudes, she must have sent something.

(Swans, Year 8 girls).

What is significant about this story is that Lizzie is tarnished through *receipt* of a dick pic and the implication that she must have sent a nude image of herself back. Indeed, it transpired that the boy had put up her details on an Instagram expose page claiming that she had sent him nudes:

Lizzie: It said… This girl is dating this fifteen-year-old and he sends nudes and she sends them back. Because people think that when boys send you nudes you send them back. It’s like an exchange thing. And then if you don’t send boys back nudes they’ll tell all their friends oh she sent those, because they don’t want to be all like…

Claire: A rejected person.

(Swans, Year 8 girls).

Here we see that the invitation for a trade creates an assumption that girls are sending nude pics back and further, that if girls refuse to trade they may be open to abusive dynamics because of the boy’s sense of rejection. Affective shame surrounded the girl (Lizzie) who had "rumours" circulated about her – and yet there is no comment about any shame or exposure experienced by the boy that sent the dick pic. This victim blaming and shaming connected to receiving the image not only impacts girls emotionally, but also makes speaking about or reporting this harassment difficult.

Further, at times girls seemed willing to take on the burden of the shame in order to protect boys’ fragile masculinity. The girls confirmed it was more likely for boys to conceal their identity in their dick pics to avoid images being spread, and that it was unusual for boys in school to send them to girls they know in person, but they then discuss one episode where these norms went wrong:

Alexus: …Some boy in our year sent it, by accident, to me and someone else.

Interviewer: By accident?

Jada: Yeah, because straight after he blocked us, because he didn’t realise he was sending it?

Interviewer: What was it like when you got the picture?

Alexus: I was confused. I didn’t know why he sent it.

Jada: I asked if anyone (else) received anything from the person. If they said yes then I’d ask if they got blocked as well, and I spoke to him about it and he said it wasn’t meant to be sent to us. He was embarrassed… It was unexpected, because I don’t really speak to him.

Alexus: It was awkward

Jada: Yeah.

(SELC, Year 10 girls)

The two girls in this episode were on holiday together and had recently posted a photo of themselves at the beach when they received the dick pic from the boy. When they did not respond to the invitational dick pic, the boy blocked them on Snapchat. The episode illustrates the networked reality of the always-connected peer group. Even when away from their peer group on holiday (or during Covid-19), the girls are still contactable through social media applications. The girls talk about feeling confused and asking other girls if they received any images and had been blocked too. Back at school they confront the boy who was “embarrassed” explaining the dick pic as meant for “someone else,” although this doesn’t gel with their discovery that he had sent the image to multiple girls. Alexus and Jada discussed the episode as “awkward” and yet collectively as a group the girls did not report the episode, instead they appear to feel sorry for and protect the boy rather than understanding or responding to the behaviour as harassment.

#### “They were my Friends”: From Disgusting, To Awkward, To Normal

A similar story was related by Year 9 participants at CLC1 who told us about an incident where two boys from school sent her a video of them masturbating:

Kathryn: So the boy on WhatsApp, he was high. Do you know who it is? Yeah, he was high so he sent me a dick pic on WhatsApp…And a video of him like wanking.

LAUGHTER

Kathryn: But I was just weirded out, I didn’t block them or anything because they were my friends, but the next day I told them what they did. And they regretted it, like a lot… Pretended to forget.

Julia: I’m pretty sure he didn’t forget that!

(CLC1, Year 9 girls)

The other girls’ question how the boys could “forget” what they had done. Here, we see the participant navigating the situation and choosing not to block them, “because they were [her] friends.” But this added social element makes the receipt of unsolicited dick pics more difficult to address, and Kathryn went on to explain: “Um, I think they just, they just really went really red and was like – fuck. They didn’t apologise though.” As the discussion continues, the full implications of girls being left to deal with these scenarios alone became clearer:

Kathryn: I think it’s more of a big deal when you know them…I think it’s worse when it’s somebody you know, because like say they are your friend, and you’ve trusted them, and you guys were really good friends, and they do that, it’s just like, it’s, personally I think it’s more of a big deal.

Julia: But it’s like because it’s your friend then like you’re already close to them, I don’t know how to explain it, they send you something that’s weird, like sexual, like weird shit, then that’s weird but like…Yeah, if they go to the same school as you then you see them every day, and it just reminds you of like what they did. So awkward.

(CLC1, Year 9 girl).

The girls discussed it as “more of a big deal” when unsolicited dick pics are sent from friends at school. They bring up the issue of trust in these dynamics, and the challenge of continuing a trusting friendship after one party sends unsolicited sexual images, saying also you cannot unsee these images and the residual awkwardness of having to go to school with these boys and see them every day. Kathryn concluded:

Kathryn: Like at first, when I first started getting dick pics I’d be like disgusted, but then I just got so used to it, and every time a dick appears on my screen, I’m like, great, again. It’s normal. So even when I got it from my friends it was like…lovely.

(CLC1, Year 9 girls)

It would be difficult to have a clearer statement of the normalisation of the ubiquitous dick pic and masculine entitlement than through this statement. The overall sense of resignation about the experience of the unsolicited dick pic is starkly apparent here. Kathryn describes the process as she moves from feeling disgusted to getting used to it, to considering it normal, which echoes the sentiments shared by many girls in our study. Throughout this conversation, we also sense the emotional labour (Hochschild, 1983) that she and other girls enact in order to protect boys by not reporting or telling anyone.

### Desirability, Differential Status of Dick Pics, and Sexual Double Standards Around Sharing

Related to the normalisation of receiving these images, we also found that some of the girls had come to associate getting dick pics from strangers and peers as a sign of desirability. This was an implication of the high scores we saw for the active Snapchat users. Carrie had lots of adults sending her Snapchat masturbation streaks, and told us this was because of her high scores on Snapchat meaning she has “loads of friends”:

Carrie: basically, obviously, my Snapchat has a massive score, which means loads of people have me as a friend, which means a load of paedophiles can send stuff to me…they just send it when I’m here to do streaks and get a higher score, but they’re just doing it the other way because they want that. And I just want streaks, because

Lou…It’s disgusting.

Carrie: If I had data (on their phone to connect to Snapchat) I could show so much like different nudes on my phone.

Sammy: You must get loads.

Carrie : Mm, I get so many.

Lou: I don’t.

Interviewer: Why must she get loads?

Sammy: Because like all of her messages, you can just tell…

Lou: She’s a really well-known person.

(CLC, 1 year eight girls).

Carrie’s privacy settings are off to enable streaks with strangers, and she is added and therefore friends with “loads of people,” meaning that, as she puts it, “peadophiles” can send her stuff. Her high visibility on social media was explained by the group as being due to the fact she was an internationally ranked competitive dancer. According to the girls’ logic, it is obvious that a high profile micro-celebrity (Marwick, 2013) status girl like Carrie would be getting lots of dick pics because she is “really well known.” 

We also found a small minority of girls actively engaged in both asking for and keeping dick pics, although this was not common. The extract below highlights how one girl sought out dick pics from a boy she only knew online on Snapchat:

Alice: I was with my friend F, and she was like, she came around to my house for a sleepover, it was like last week, like previously she’d already been talking to this boy who lived in Ireland, on Snapchat, just for like the sake of it, and then she was like oh wouldn’t it be funny if we tried to get nudes off him and then not send them back. She was texting him, being like oh if you send them I’ll send you stuff back, but she was like I’m not gonna send him anything, isn’t that funny. And then he’d be like but don’t screenshot it, because I’ve had it before… and I got really upset when they never sent any back, and they screenshot it. And she was like don’t worry, I will, I will, and then he sent them, and she said I’m not satisfied, send me another one, and then he sent her another one, and then she just left him and turned her mobile data off and just headed for the rest of the day. And she was like oh I’m never gonna meet him, he lives in Ireland.

(Swans, Year 8 girls)

**Fig. 6:**
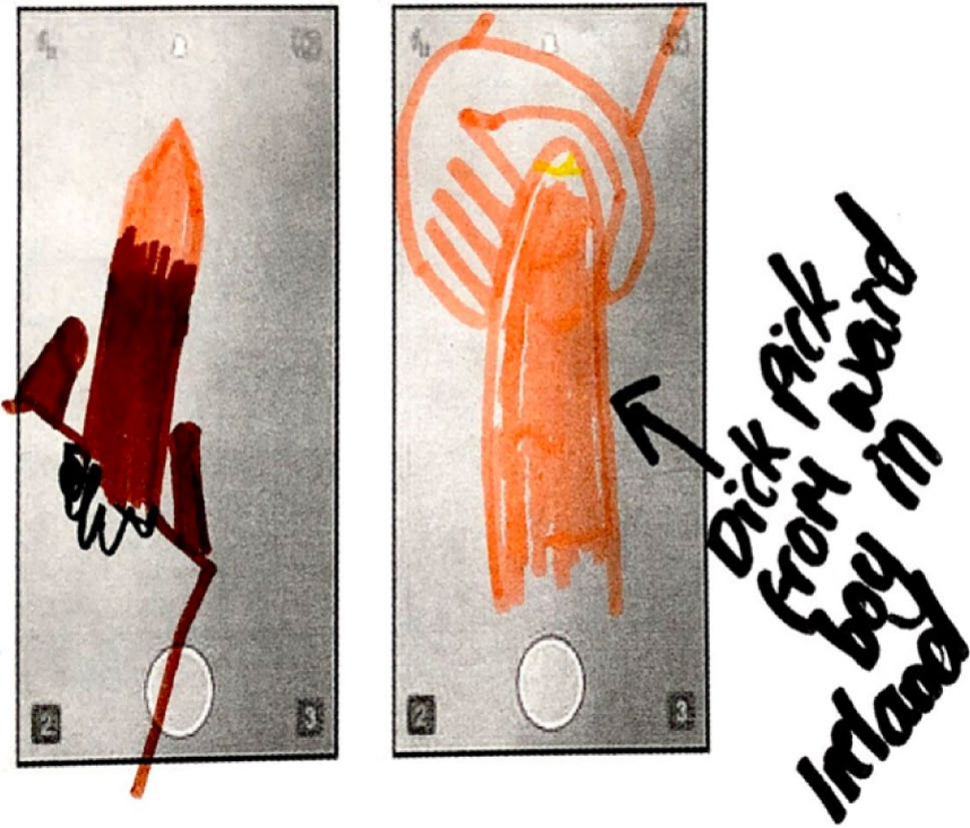
Drawing of Snapchat Private Message: “Dick pick from weird boy in Ireland” (Swans, Year 8 girls)

Figure 6 depicts a drawing of a dick pic from the boy in Ireland discussed by Alice. This example demonstrates how one girl catfished a boy, or procured nude images from a boy via Snapchat through trickery—the implication that it was part of a trade from her, but she did not send one back. Here the girl is positioning herself as a reward for the boy, that she is desirable and sexually experienced enough to get nudes from boys and telling her friend to gain status. Another girl was described as keeping a dick pic after a relationship ended:

Daphne: Mm, she’s broken up with A, but she still keeps his dick pics on her phone Yeah, she was going through her camera roll and I said what the hell? And she said oh look, it’s A’s dick. I was like OK. It’s like you’re not going out with him anymore, you can delete that. But she just keeps it on there.

Alice: It sounds kind of weird, but like a trophy, you know. She dumped him because he made out with L and D. She texted him, and was like, she showed me the screenshot, she was like I’m too good for you, you don’t, I’m not with you anymore. And then like I feel like deep-down she still wishes she was with him. I feel like if he didn’t do that she’d still stay with him. Oh like I’m so popular, I’ve got like a dick pic, or I’ve got this…

Claire: So then you kind of have power over them, because you know…you can expose them, and it’s power.

(Swans, Year 8 Girls)

The girls discussed keeping a dick pic as a trophy and as leverage, which the girl may be able to harness for power over the boy because the girl can expose him. But ultimately that does not tend to happen; rather we can see there are complex dynamics of shame and pity for the girl around her keeping the dick pic, discussed as “weird.” Other girls confirmed that they would be unlikely to publicly share dick pics first because they were usually non-consensual in the first place, but also because as we have discussed, confirming receipt of dick pics could be shameful for girls and tantamount to admitting they sent nudes back. Girls were also worried that they would get in trouble for telling on boys (being called a "snake" or snitch) or socially shamed if they shared dick pics with others:

Interviewer: So why are you worried about being called a snake, but boys are not worried about it?

Camilla: Boys it’s like a trophy, for girls it’s like shameful to share.

Kate: For boys it’s kind of like, it heightens them up, they are like oh I got a girl…Normalised with boys to like behave that way, I think.

(Swans, Year t10 girls)

**Fig. 7:**
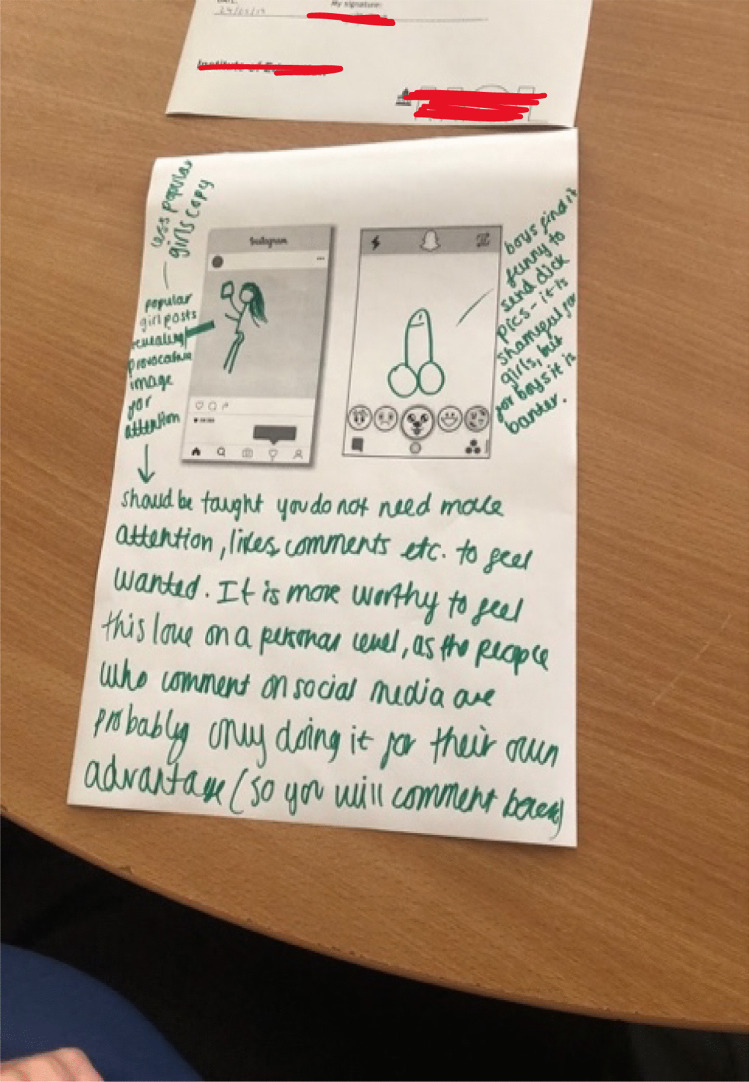
Drawing of girls body and boys dick pic and tips on needing "male attention": “Boys find it funny to send dick pics, girls find it shameful” (Swans, Year 9 girls)

This exchange signals a heterosexual cisgendered exchange economy with differential rewards for boys and girls (see Fig. 7). Although girls can certainly ask for and keep dick pics, it was not as likely for them to openly discuss doing so as these practices come with certain risks of being slut shamed. There does not seem to be the same risk of shame for boys who send dick pics or who collect girls’ nudes as “digital trophies” (Berndtsson & Odenbring, 2020). But dick pics do not work as trophies for girls in the same ways. Indeed, in Fig. 7 the girls compared images of girls’ whole bodies to garner male attention compared to the zoomed-in penis image, which they position as a form of male banter directed towards girls. They note that boys having nudes of girls is a currency which “heightens” them, but state again that it is shameful for girls to share boys’ nudes. The quote and image effectively sum up the sexual double standards endemic to dick pick practices. The drawing also includes comments that challenge the value of these pics and questions the need for “attention,” and “love,” or and feeling “wanted” by boys through the exchange of these pics.

### Resisting the Normalisation of Unsolicited Images: Re-animating the Dick Pic Through Drawings

#### Humour and Critical Engagement

Across all seven research schools, we found an overwhelming proportion of participants accepted coercive, victim-blaming, and pressuring practices on social media as the norm because they had never examined these practices within frameworks that positioned this behaviour as harassment. However, we also found that the drawings provided a space to dwell upon and sometimes disrupt the episodes that had been recounted. For instance, at Swans the girls talked critically about homosocial masculinity practices on social media, including the types of images taken and the mode of sending of what they called “provocative” images to girls:

Interviewer: You think sending a picture of their part has now become a part of banter?

Camilla: Yeah.

Kate: Yeah.

Lily: They think it’s funny.

Camilla: They like have a laugh.

Kate: They have a competition, like how many responses you can get.

Lily: They’ll be hanging out.

Ash: Yeah, and they’ll all send a picture at the same time, and sometimes it’s so bait because you’ll get pictures from like three boys at the same time.

Camilla: Yeah, you know that they’re all friends.

Kate: You know they are all together.

Camilla: If they are pictures on the same lines you know that they’re having a competition.

Kate: and then they’ll just see, sometimes they’ll all send it to one specific girl, and see who gets the response [from a girl] the fastest.

Lily: it’ll just be a picture of a bulge under clothes or something.

Camilla: At the same time. All just photographing their junk.…It is weird to think about, it’s like the more attention they get the cooler they are, they try and make, I’ve got more replies than you have, or whatever.

Kate: Gross. It’s just this really toxic idea of what’s masculine.

(Swans, Year 10 girls)

**Fig. 8:**
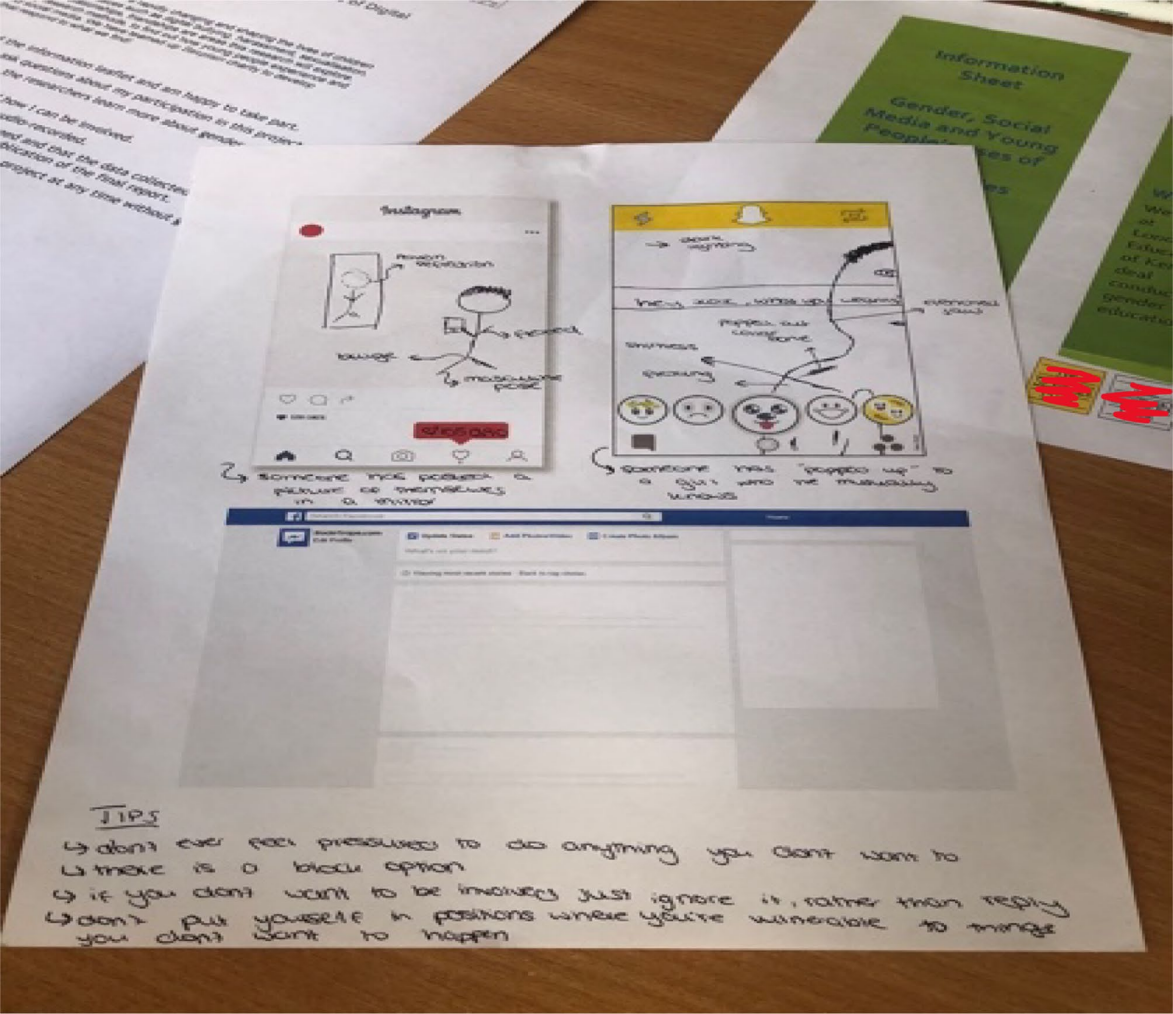
Drawing of Boy’s Bulge Selfie, and tips, “don’t ever feel pressure” and “there is a block option” (Swans, Year 10 girls)

**Fig. 9:**
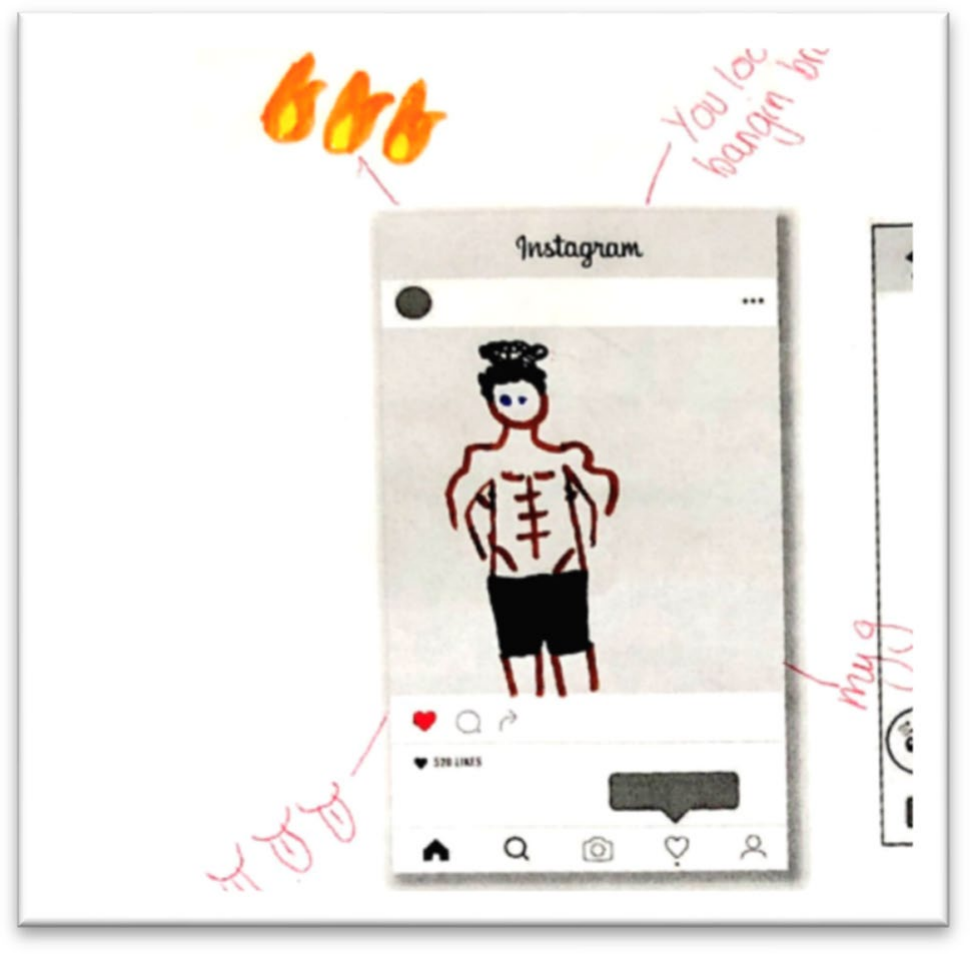
Drawing of an Instagram image of boy’s v-line: “You look bangin’ babe” (Swans, Year 10 girls)

The girls discussed the practice of boys taking pics of their “junk” together as a group and sending it around to a network of girls trying to get a response as a “competition.” The girls challenge this behaviour as merely “banter,” and describe it as a “really toxic idea of what is masculine.” They are invoking a current buzzword of *toxic masculinity* (Harrington, 2020), and are recognising the power imbalances with boys sending out uninvited images as part of homosocial lad banter, which may serve as a reward for boys in the form of being seen as “cooler” by other heterosexual boys (see also Ravn et al., 2019). The art-making process together with the discussion space opened up some moments of affective hilarity as girls began drawing penis “bulges” and “V-lines” indicating the pelvic region in Figs. 8 and 9.

Through the drawings and group discussion, then, the girls sometimes collectively challenged the normalisation of online sexual harassment via unwanted images, laughing and using sarcasm, such as that found in the humorous caption in Fig. 9, “you look bangin’ babe,” echoing the findings of Vitis and Gilmour (2017) which noted how poking (no pun intended) fun at dick pics offers a way of resisting feelings of victimisation around these images (see also Ringrose & Lawrence, 2018).

**Fig. 10:**
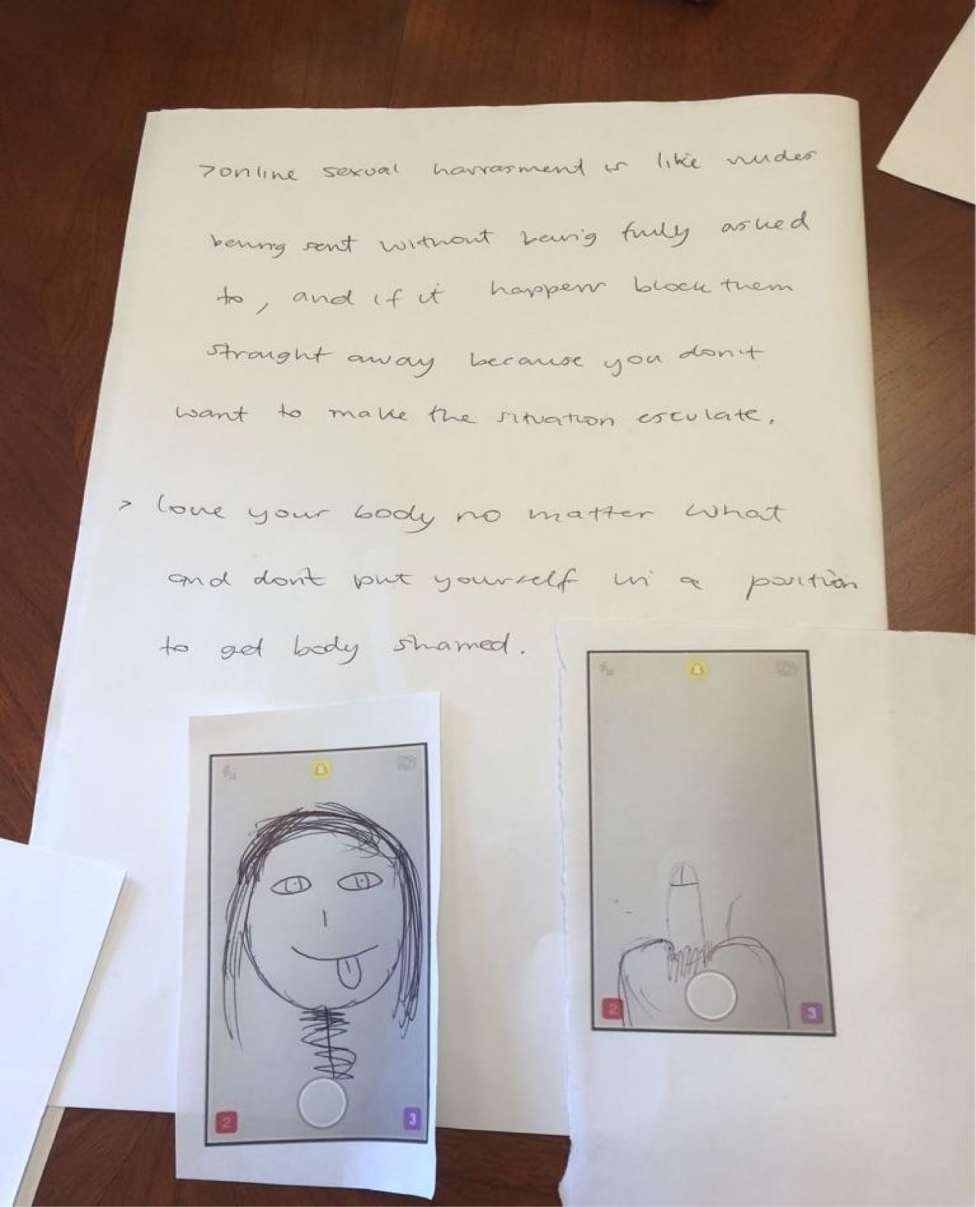
Drawing of “birds eye view” dick pic and tips on “online sexual harassment harassment” “nudes being sent without being fully asked…” (NELA, Year 10 girls)

Our discussions in several other groups moved further into a critical analysis of gendered power dynamics by raising the idea that what girls were experiencing through mass dick pic bombardment was a form of digital sexual harassment (Powell & Henry, 2017). Girls at NELA said they had never had occasion to discuss their experiences of dick pics because they told us it was not a part of any of their sexuality education. In the drawing session, they documented ambivalent feelings of being sent something “without fully being asked,” which begins to articulate an awareness of sexual consent and lack thereof, as seen in Fig. 10.

We can see in the description of the images they drew that the girls were explicitly discussing how being sent dick pics is a form of online sexual harassment, and they also became more aware of reporting strategies through our discussion. Likewise, the SELC Year 10 girls who had received Snapchat streaks of “grown men” masturbating, and the “accidental” dick pic from their peer, went even further during the drawing session by directly confronting these unequal dynamics. They raised the point that girls and women now have feminism to “stand up for girls and women,” and they went on to disrupt the new normal of the unsolicited dick pic:

Jada: Can I say something completely different?…They think it’s normal.…yeah, it’s normal, or they didn’t do anything, and that is sexual assault! But most teenagers don’t know that, so they don’t do anything about it, and they just leave it.

Alexus: And they’re feeling bad.

Interviewer: That’s really useful and important, have you been taught about sexual assault in your sex education?

Jada: I don’t know what every single sexual assault is, I don’t know what you could define it as.

Rhianna: My school did sex education once and I missed it, so I’ve never had sex education.

Interviewer: OK, and this is really interesting, and it’s not unrelated, because do you think that some of the things that happen online are a form of online sexual harassment?

Jada: Yes.

(SELC, Year 10 girls)

**Fig. 11:**
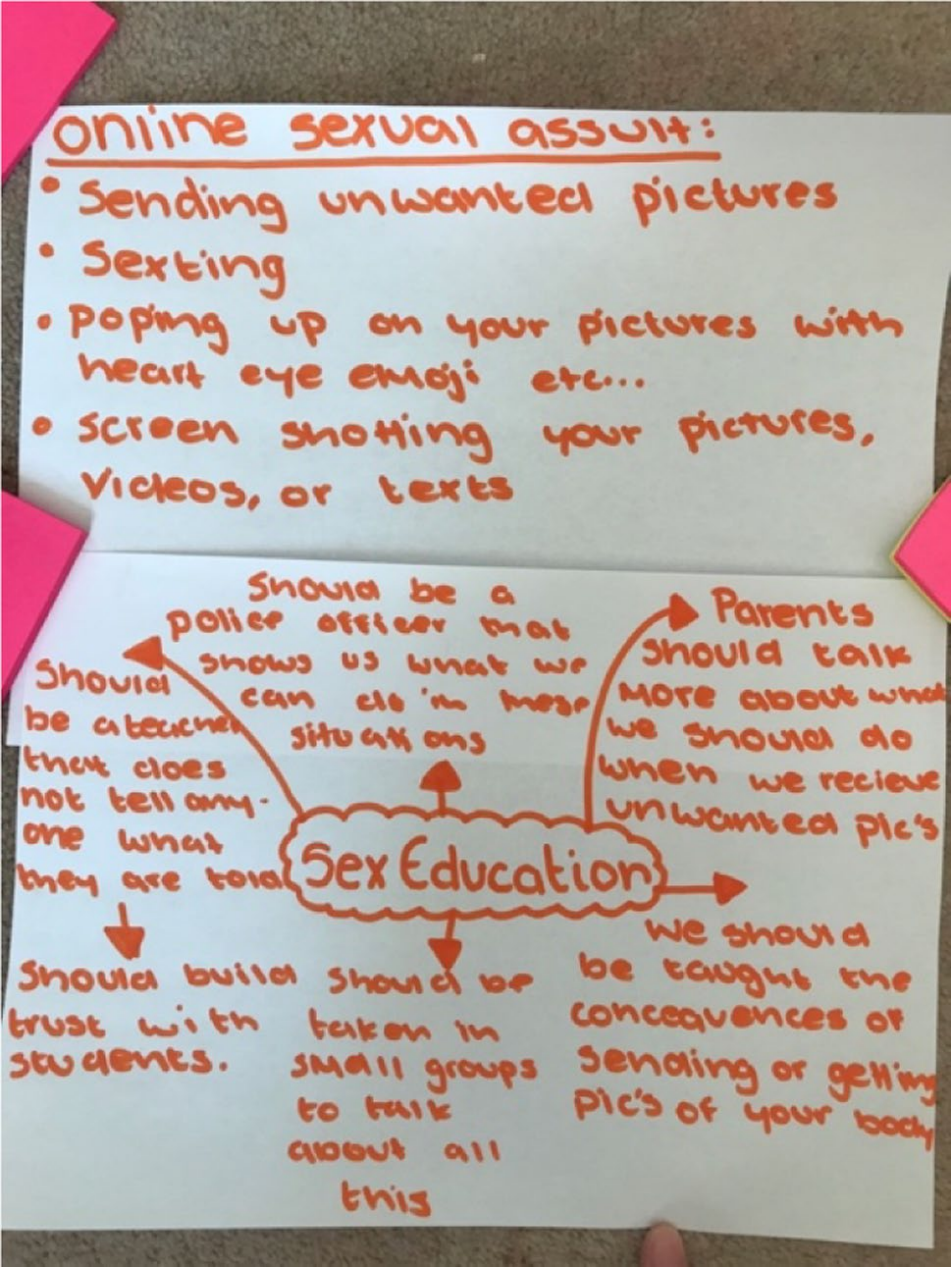
Drawing/ Mind Map Covering Online Sexual Assault and Sex Education (SELC, Year 10 girls)

In this excerpt, without any prompting, a participant says that because sending unwanted images is so normalised, it is not understood as a form of assault. They again relate never having learned about online forms of sexual assault or harassment in their sex education at school and they laid out the problem and recommendations in their mind map (see Fig. 11).

The girls’ drawings build on the discussion of sexual assault and offer explicit instructions about what needs to change in sex education. These examples show the power of the drawing sessions to create spaces for critical reflection where young people can point out inequity and offer important ideas about how to do things differently.

## Discussion

In this study, we explored how girls are bombarded with unsolicited dick pics from both unknown men and boys in their peer groups, particularly on the platform Snapchat. We discussed a range of strategies that girls develop to manage the receiving of unwanted dick pics, which involved primarily ignoring and blocking them, but rarely reporting them. Across all seven school sites, we found a strong trend amongst most of the girls whereby they become used to the pics and resigned about the inevitability and “ubiquity” of receiving dick pics, as shown in previous research (Mandau, 2020; Ricciardelli & Ajordan, 2018, p. 563). We also found that while the unsolicited dick pic from unknown users remains more common, girls were more easily able to ignore, block and delete “randoms” and “paedos” than the pics sent from known boys within their peer group. Unsolicited dick pics from peers at school were described as a “big deal” and difficult to navigate. Some of the dick pics were transactional and sent as part of pressuring girls to send nudes back, which confirms earlier research on the prevalence of pressured sexting (Englander & McCoy, 2016).

Reporting the unwanted dick pics from peers to the social media platform, school or adults was difficult for multiple reasons. Boys tend to send faceless dick pics to conceal their identity. Girls could not save the images from Snapchat because the platform notifies a screen save to the user, which could be seen as encouragement or could also put the girls at risk of further harassment from boys since girls faced victim blaming around reporting their peers, such as being called a snake. Reporting dick pics is also not an option for some girls because they want to protect what is perceived to be a vulnerability in the boys sending dick picks. We observed that some girls felt compelled to avoid bringing further embarrassment or awkwardness to the situation, which made the girls responsible for managing their own victimisation in ways similar to Amundsen’s (2020) findings with adult women. Also, like Amundsen, we found that most girls did not identify unwanted dick pics as harassment.

Not all girls shared the same experience of weary resignation; we also found that some girls wanted unsolicited dick pics because these marked them out as desirable. This was particularly the case for girls with high social media visibility and popularity, who wished to make their sexual status as desirable known to peers. As reported in other research that has explored consensual sexting (Naezer & van Oosterhout, 2021; Ravn et al., 2019; Setty, 2019b), we also found a small minority of girls discussed asking for and keeping dick pics. The girls recounted one experience whereby a girl catfished a boy by promising nudes of herself if he sent some to her first, and then ghosted the boy after he sent the pics. We also found episodes of consensual sexting whereby a girl was reported to have kept a dick pic after a break up as a trophy. However, in this narrative, the girl was then sexually stigmatized for keeping the image, so it did not serve as a form of currency or evidence of sexual kudos in the same way that nudes of girls tend to be celebrated amongst heterosexual boys (Ringrose & Harvey, 2015; Salter, 2016a). Like Setty’s (2019a) research, girls’ faced the possibility of being shamed for being known sexters. Moreover, being a known recipient of dick pics was often interpreted as evidence of girls sending nudes back to boys, even if that was not the case. This indicates the ongoing prevalence of sexual double standards around boys’ and girls’ nudes (Setty, 2019b). Overall, girls are not able to use images of boys’ bodies as trophies in the same ways that boys use girls’ nudes since receiving dick pics can become a source of sexualised shame for girls.

### Limitations and Future Research Directions

Whilst the focus groups were an excellent vehicle for generating discussion on the research topic, focus group methodologies may also present a limitation. We found very high rates of girls reporting that they had received unwanted dick pics as well as most of the girls reporting they had experienced pressure to send nudes. One of the possible limitations of focus groups is that they encourage uniformity of responses since participants perform their gender identity (e.g., ideal femininity) to the wider group (Nayak & Kehily, 2006). One way that we felt we addressed some of the limitations of the focus groups was using arts-based methods, which offered another rich source of data to explore the experiences of digital image sharing. In the drawing part of the workshops, the young people were instructed to draw their experiences and offer tips for change. The drawings were individual although conducted in the group setting. There were often periods of silence and introspection while drawing and we found this reflection period was key in providing a space of critical engagement. The drawings, top tips and mind maps did something different than the talk, enabling young people to dwell upon some of their experiences and articulate (write down) what they would like to see change. In some of the drawing sessions, girls were critical of and poked fun at some of the performative masculinity practices of boys’ sending around images of their “bulges,” “junk” and other “provocative” poses. We also found some girls discussed the need for more education around digital sexual assault and abuse and sketched mind maps around what should change to provide the information and tools young people need to recognize and combat problematic and non-consensual social media image practices.

Participatory research that positions young people as change agents and asks their views has been long recognized as important from a child’s rights perspective (Renold et al., 2018; Setty, 2019a). Drawing social media experiences also helps to move beyond some of the ethical limitations of collecting sensitive, private digital data from young people (Facca et al., 2020). Further research using arts-based methods such as drawing or collaging that encourages memory work to explore online experiences would prove interesting as it provides different forms of engagement beyond talking about social media practices.

### Practice Implications

Our findings demonstrate that specific affordances of platforms, such as Snapchat, have been central in the rise of unsolicited dick pics. Snapping is a practice of taking a quick snap and sending it and is non-consensual from the outset, and the ephemerality of disappearing media limits capacities for reporting (Charteris et al., 2018; Handyside & Ringrose, 2017). Platform specific guidance is needed on privacy settings and reporting, but in ways that do not re-victimize girls who are sent images or make girls’ responsible for managing their own harassment.

We found reporting was also limited because young people often did not understand practices of non-consensual dick pics as harassment. Therefore, we also need to move away from the type of youth sexting guidance we outlined in the context section, which focuses on all youth sexual images as criminal and offer youth abstinence messages around sexting, which have neglected the complexity of gendered power relations around images and platform specificities. Criminalising all nude images of under 18 s makes it difficult to distinguish when and how practices of image exchange become harassing and abusive. It also means, for instance, that girls could be found in possession of child pornography if they report dick pics they have received from their peers, which would entirely fail to recognize the non-consensual conditions under which they may have been sent these images.

We advocate for a shift to understand all non-consensual dick pics as forms of image based sexual harassment (McGlynn & Johnson, 2020) as a starting point. Being able to identify non-consensual practices as harassment is only part of the solution, however. Setty (2019b) emphasises the need for a collectivist digital sexual ethics amongst young people. This entails developing a greater awareness of gender inequalities that underpin the differential treatment of nude images of girls’ and boys’ bodies in the wider public domain (Salter, 2016b). Securing images of girls’ bodies continues to be a source of recognition and reward for boys; whereas dick pics can both be used as a form of harassment and girls can also be shamed if they are known recipients, whether these images were asked for or not. It is therefore critically important to disrupt these sexual double standards. As we indicated through discussion of the drawing methodology, young people need to be offered educational spaces for reflection, humour and resistance to norms of girls and women as “vulnerable and powerless” in the face of dick pics; enabling the potential for forging collective techniques of disruption (Vitis & Gilmour, 2017, p. 348).

## Conclusion

In this article, we have sought to highlight the normalization of unsolicited dick pics sent to girls aged 11–18. Our research confirms and extends earlier research that suggested dick pics have become a ubiquitous part of youth digital sexual cultures (Ricciardelli & Adorjan, 2018). We found our participants for the most part lacked a framework to understand these experiences as harassment, and they did not typically report these practices. Instead, they ignored or blocked the senders, but doing so was more difficult when the sender was a known boy from the peer group at school. We discussed how our drawing methodology enabled girls to break through some of the barriers of masculine “sexual entitlement,” (Hayes & Dragiewicz, 2018, p. 115) in image sharing practices, repositioning dick pics as toxic, harassing and even a form of assault; all without explicit promptings; showing young people have the tools for critical thinking when encouraged to explore and offer what they think should change. We advocated for a shift in terminology to understand youth digital sexual image sharing, replacing victim-blaming narratives and abstinence messages derived from the criminalisation of all youth sexual images, to a focus on how and when image sharing and receiving are non-consensual, harassing and abusive. We hope that by explicitly adopting the concepts of online sexual harassment (Vitis & Gilmore, 2017) and image based sexual harassment (McGlynn & Johnson, 2020) that educators, parents and young people will be better able to grasp the nuances of when youth online image sharing practices become non-consensual and harmful, which will enable a disruptive shift and positive transformation in youth digital sexual cultures.

## Data Availability

The datasets generated during and/or analysed during the current study are not publicly available due to reasons of confidentiality and anonymity in in the subject area and majority of the data are with under 16-year-olds. Anonymized transcripts and drawings can be made available from the corresponding author on reasonable request.

## Declarations

### Research Involving Human and Animal Participants

This research involves human participants and ethical approval was attained by The University of Kent Research Ethics Advisory Group (REAG)
